# Corrigendum

**DOI:** 10.1111/cns.13900

**Published:** 2022-06-25

In Zhang et al.,^1^ the authors noticed that the same figure was accidently presented in Figure 1K and L.

**FIGURE 1:**
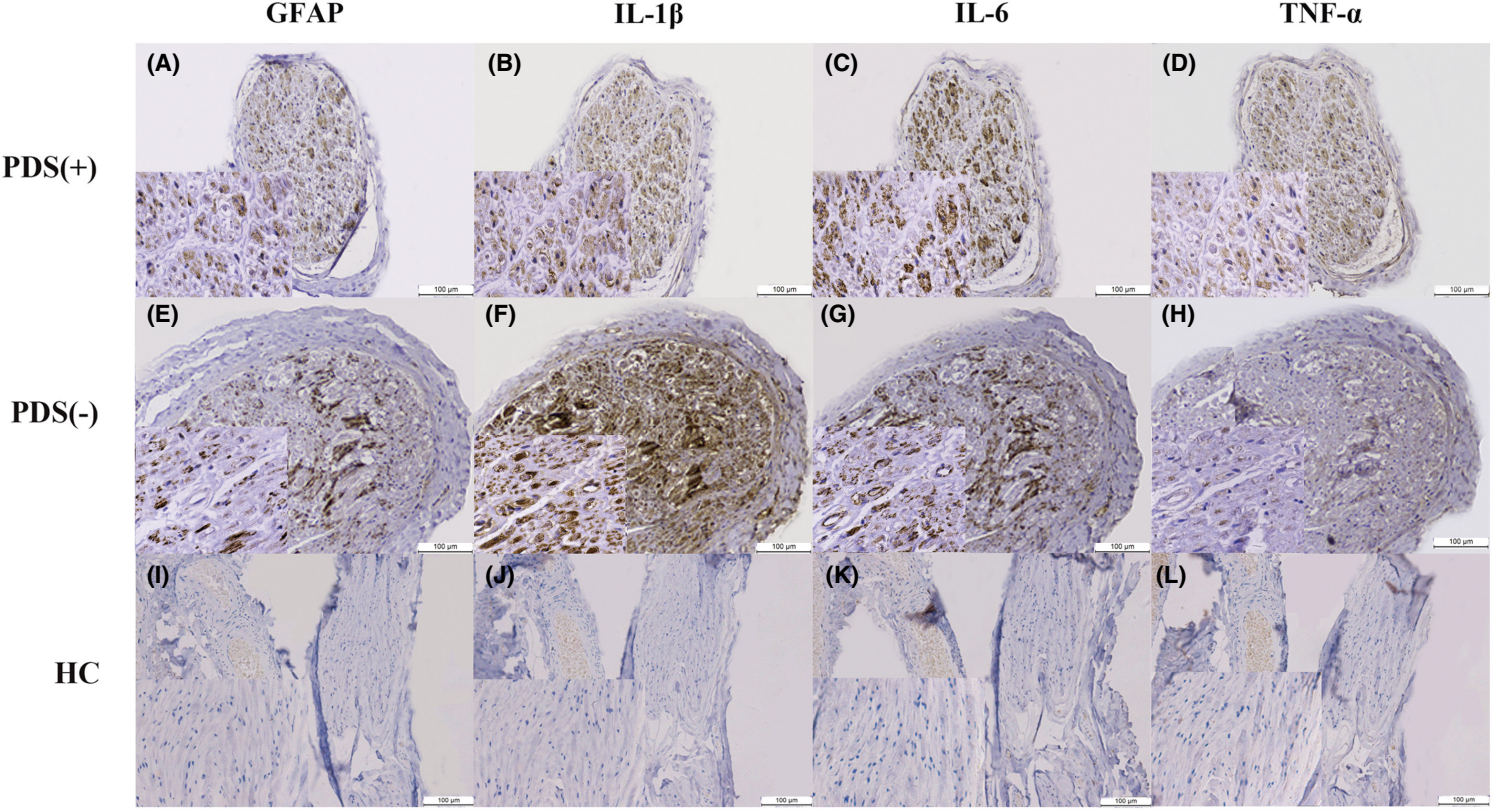
GFAP, IL‐1β, IL‐6, and TNF‐α in serial sections of sural nerve. The brown inclusions inside the nerve bundles were positive immunoreaction to GFAP (A, E, I), IL‐1β (B, F, J), IL‐6(C, G, K), and TNF‐α (D, H, L) in a 60‐y‐old female PD patient with sensory disturbances (A–D) (case PD patient 11 in Table 1), a 71‐year‐old female PD patient without sensory disturbances (E–H) (case PD patient 10 in Table 1) and a 63‐year‐old male control without neuropathy (I–L) (case Control 1 in Table 1). Original magnification was 100×. Insets were all set at magnification (400×). Abbreviations: GFAP, glial fibrillary acid protein; HC, healthy controls; IL‐1β, interleukin‐1‐beta; IL‐6, interleukin‐6; PD, Parkinson's disease; PDS (−), PD patient without sensory disturbances; PDS (+), PD patient with sensory disturbances; TNF‐α, tumor necrosis factor‐alpha

The correct Figure 1 is given below:

The Author's apologize for this error.
